# Carbon nanotubes as a nitric oxide nano-reservoir improved the controlled release profile in 3D printed biodegradable vascular grafts

**DOI:** 10.1038/s41598-023-31619-3

**Published:** 2023-03-22

**Authors:** Fatemeh Kabirian, Pieter Baatsen, Mario Smet, Amin Shavandi, Petra Mela, Ruth Heying

**Affiliations:** 1 Department of Cardiovascular Sciences, Cardiovascular Developmental Biology, KU Leuven, Leuven, Belgium; 2 VIB-KU Leuven Center for Brain and Disease Research, Department of Neurosciences, KU Leuven and EM-Platform of VIB Bio Imaging Core at KU Leuven, Leuven, Belgium; 3 Department of Chemistry, Polymer Chemistry and Materials, KU Leuven, Leuven, Belgium; 4 École Polytechnique de Bruxelles, 3BIO-BioMatter, Université libre de Bruxelles (ULB), Brussels, Belgium; 5 Medical Materials and Implants, Department of Mechanical Engineering and Munich Institute of Biomedical Engineering, TUM School of Engineering and Design, Technical University of Munich, Garching, Germany

**Keywords:** Implants, Drug delivery, Drug delivery, Tissue engineering, Translational research

## Abstract

Small diameter vascular grafts (SDVGs) are associated with a high failure rate due to poor endothelialization. The incorporation of a nitric oxide (NO) releasing system improves biocompatibility by using the NO effect to promote endothelial cell (EC) migration and proliferation while preventing bacterial infection. To circumvent the instability of NO donors and to prolong NO releasing, *S*-nitroso-*N*-acetyl-d-penicillamine (SNAP) as a NO donor was loaded in multi-walled carbon nanotubes (MWCNTs). Successful loading was confirmed with a maximum SNAP amount of ~ 5% (w/w) by TEM, CHNS analysis and FTIR spectra. SDVGs were 3D printed from polycaprolactone (PCL) and coated with a 1:1 ratio of polyethylene glycol and PCL dopped with different concentrations of SNAP-loaded matrix and combinations of MWCNTs-OH. Coating with 10% (w/w) SNAP-matrix-10% (w/w) SNAP-MWCNT-OH showed a diminished burst release and 18 days of NO release in the range of 0.5–4 × 10^–10^ mol cm^−2^ min^−1^ similar to the NO release from healthy endothelium. NO-releasing SDVGs were cytocompatible, significantly enhanced EC proliferation and migration and diminished bacterial viability. The newly developed SNAP-loaded MWCNT-OH has a great potential to develop NO releasing biomaterials with a prolonged, controlled NO release promoting in-situ endothelialization and tissue integration in vivo*,* even as an approach towards personalized medicine.

## Introduction

Nitric oxide (NO) is an endogenous diatomic molecule that regulates many physiological responses such as acceleration of endothelialization and tissue integration, inhibition of platelet adhesion/aggregation and neutralization of pathogenic bacteria^1^. The combination of NO in the design and development of biomaterials may improve the biological functionality of these materials intended for tissue regeneration and healing applications.

Therefore, an important research goal in biomedical cardiovascular engineering is to design NO-releasing biodegradable small diameter vascular grafts (SDVGs, < 6 mm) that can release NO for several weeks, required for complete endothelialization^2^, in the physiological range of 0.5 to 4 × 10^–10^ mol cm^−2^ min^−1^^1^.

*S*-Nitrosothiols (RSNOs) such as *S*-nitroso-*N*-acetyl-d-penicillamine (SNAP) are a class of biocompatible NO donors that in response to heat, moisture, or light radiation release NO by cleavage of the S–N bond^3^. However, *S*-nitrosothiols have low in vitro or in vivo stability and therefore RSNOs have been incorporated within polymeric reservoirs such as polyurethane, polyethylene glycol (PEG)-polycaprolactone (PCL)^4^, silicone^5,6^, silk fibroin^7,8^.

Multi-walled carbon nanotubes (MWCNTs), are a class of nanoparticles with excellent thermal and chemical stability and a large cavity area suitable for loading of drug molecules including NO donors^9^. Aggregation due to van der Waals interactions and subsequent reduction of the effective loading capacity is a major limitation of MWCNTs for drug delivery applications. Unmodified MWCNTs form non-dispersible nanotube bundles that induce apoptosis of human cells^10^. However, MWCNTs functionalized with hydrophilic hydroxyl groups (MWCNT-OH) allow multiple hydrogen bonding and dipole–dipole interactions resulting in reduced aggregation and better dispersibility in polar solvents than non-modified MWCNTs^9,11^.

In the in-situ tissue engineering approach, an implanted acellular graft should provide mechanical support for tissue regeneration, be degraded gradually while being replaced by native tissue in vivo and ideally contain molecules to encourage host cells’ migration into the graft.

In our previous studies, we showed that incorporation of 10% (w/w) SNAP in a PEG-PCL matrix with a PCL topcoat resulted in a physiological release of NO (14 days) which was associated with the limitation of a high burst release^4,12^. Although a longer physiological release of NO can potentially support the early adaptation of grafts in vivo, loading of a higher concentration of SNAP in this polymeric matrix leads to a longer burst release. Therefore, the advantages of nanocarriers in controlled release systems as a high carrier capacity have been considered. In this study, we hypothesize that MWCNTs loaded with SNAP and incorporated within the same polymeric matrix and coated in the lumen of 3D printed SDVGs could prolong the NO release in the physiological range without enhancement of the burst release. This NO-releasing SDVGs could then improve endothelial cell migration and proliferation while creating an antibacterial environment. In this work, we designed and developed a novel controlled release system of NO by loading of SNAP in MWCNTs. The effect of parameters as solvent polarity, concentration of MWCNTs and MWCNT functionalization were evaluated by CHNS elemental analysis and Fourier transform infrared spectroscopy (FTIR). The optimized loaded MWCNTs were incorporated into a polymeric matrix and coated on biodegradable 3D printed SDVGs for further characterizations of the NO release profile. Biological studies investigating antibacterial properties, cytocompatibility, endothelial cell (EC) morphology, proliferation and migration and platelet adhesion were performed.

## Results and discussion

**Figure 1:**
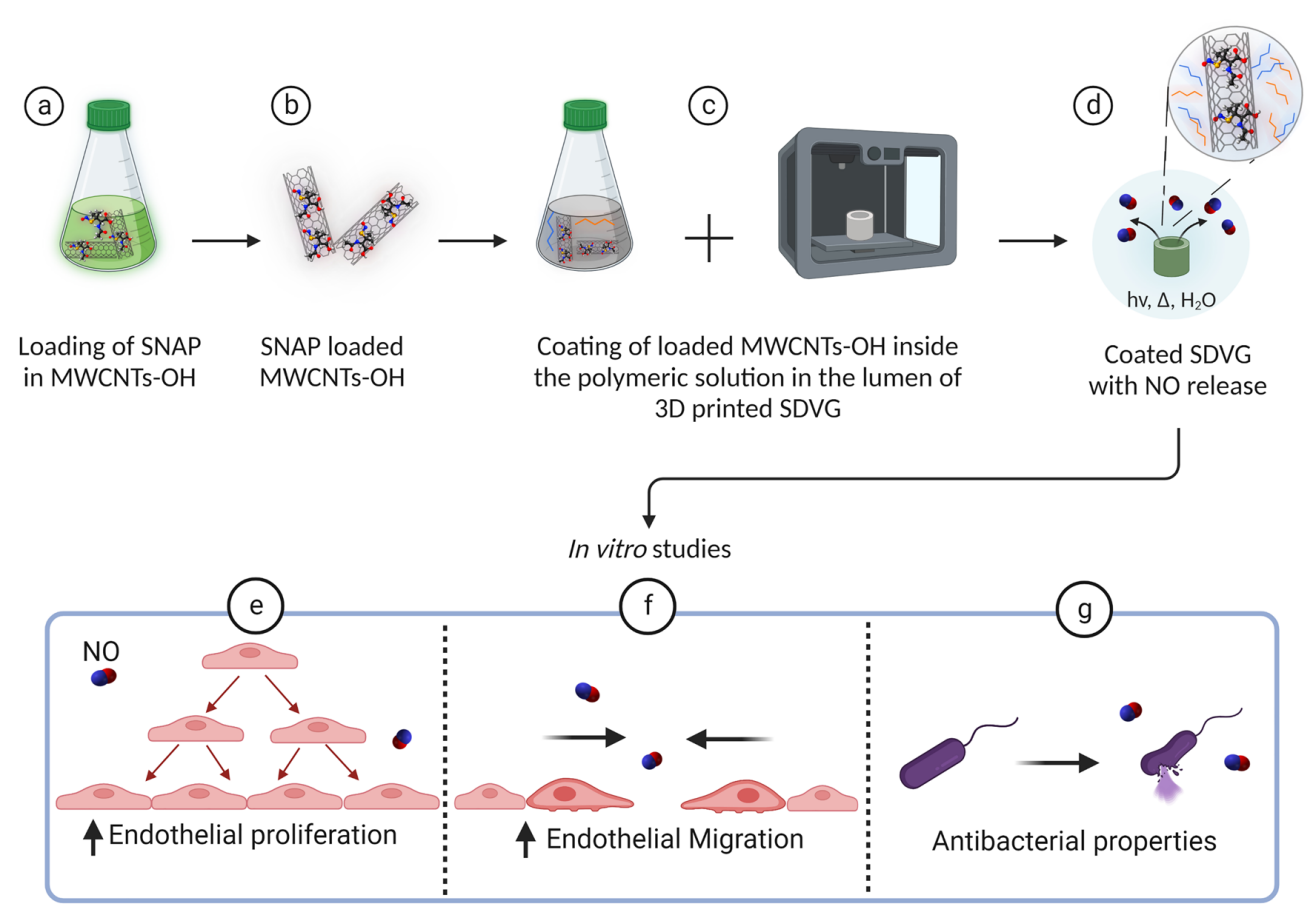
Schematic representation of the preparation of NO-releasing vascular grafts (SDVGs) and their biological properties in vitro. The upper panel shows (**a**,**b**) the loading of SNAP in MWCNTs-OH, (**c**) coating of them inside the lumen of 3D printed SDVG and (**d**) NO release from the coated SDVG. The lower panel indicates the biological properties of the NO releasing graft such as increase in EC proliferation (**e**) and migration (**f**) and antibacterial properties (**g**).

In this study, for the first time, the loading of SNAP on MWCNTs-OH and its combination into a polymeric matrix as a coating is reported. Although this innovative NO controlled release system is applicable for different medical devices, we elaborated it in SDVGs (Fig. 1). SDVGs display a high incidence of failure due to poor endothelialization and a subsequent risk for bacterial infection^4^. Therefore, an NO-releasing coating can promote the next generation of SDVGs with improved clinical performance.

### 3D-printing and coating of SDVGs

**Figure 2:**
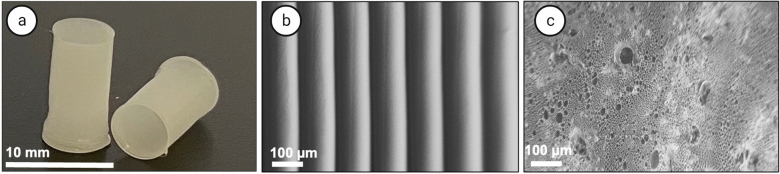
Morphology visualization of SDVGs with and without the coating. Macroscopic image of 3D printed tubes (**a**) and SEM images of 3D printed tube (**b**) and the coated tube with 10% (w/w) SNAP-matrix—10% (w/w) SNAP-MWCNTs-OH (**c**).

3D printed conduits fabricated from PCL, using an FFF printer were shown in Fig. 2a. The SEM images reveal the homogeneous layer morphology of the layer-by-layer printed grafts (Fig. 2b). The intraluminal morphology changed after coating with 10% (w/w) SNAP-matrix-10% (w/w) SNAP-MWCNTs-OH to a porous structure (Fig. 2c) due to the evaporation of solvent, remaining the voids which can encourage cell infiltration while still the outer layer is non-porous to prevent blood leakage. The advantages of the 3D printing approach such as the potential for preparation of customized implants, reproducibility, accuracy and cost-effectiveness has been previously applied for fabrication of SDVGs from polylactic acid (PLA)^12^, PCL^13^ and poly(propylene fumarate)^14^.

### SNAP loading of MWCNTs-OH

The results demonstrate that with 25 mg MWCNT or MWCNTs-OH and 10 mg SNAP, MWCNTs-OH can store a higher amount of SNAP (5.00 ± 0.67%N, Supplementary Table) compared to non-functionalized MWCNTs (2.07 ± 0.84%N). A plausible explanation for this result is the lower tendency of aggregation in MWCNTs-OH which can increase the loading capacity. Therefore, MWCNTs-OH was used for further investigations in the current study.

The effect of solvent polarity was investigated by SNAP loading in polar and non-polar solvents. The amount of SNAP loading in MWCNTs-OH in polar solvents were significantly lower (0.37 ± 0.01 and 0.30 ± 0.05%N for THF and MeOH) than in non-polar solvent (5.00 ± 0.68%N for toluene). This finding as expected is due to the polarity of SNAP molecule and a subsequent great affinity for polar solvents. Therefore, in the non-polar solvents such as toluene, SNAP molecules have a greater affinity to MWCNTs-OH than solvent resulting in a higher loading of SNAP in MWCNTs-OH.

The SNAP loading capacity of MWCNTs-OH was also studied by loading of a constant amount of SNAP (10 mg) in various amounts of MWCNTs-OH (25, 50 and 100 mg). The results indicate that with 10 mg SNAP and 25 mg MWCNTs-OH, the maximum amount of 5% (w/w) SNAP would be loaded. This condition was selected for all the follow-up experiments.

To explain the loading process of SNAP in MWCNTs-OH, as it was shown by Khalifi et al., there is an energy barrier at the edges of the CNT, which hinders the adsorption of drug molecule. Once the drug molecule passes through this energy barrier, it displaces toward the middle parts, as the adsorption energy at the middle of the CNT is much lower than the edges^15^. Stirring the drug molecules with MWCNTs-OH can overcome the required energy barrier at the nanotube edges. It will also leads to a slower release profile, as it allows for a stable adsorption of drug molecules by the middle parts of the MWCNTs^16^.

Various nanoparticles are used to develop NO controlled release systems. For example, *N*-diazeniumdiolates as a NO donor was encapsulated in the liposomes and NO release was prolonged up to 24 h^17^. In another study, SNAP was encapsulated in silk fibroin nanoparticles and NO release was prolonged over 24 h^8^. In this study, MWCNTs-OH were used as nanocarriers for SNAP to develop the controlled release system of NO.

### Characterization of the SNAP loaded MWCNTs

**Figure 3:**
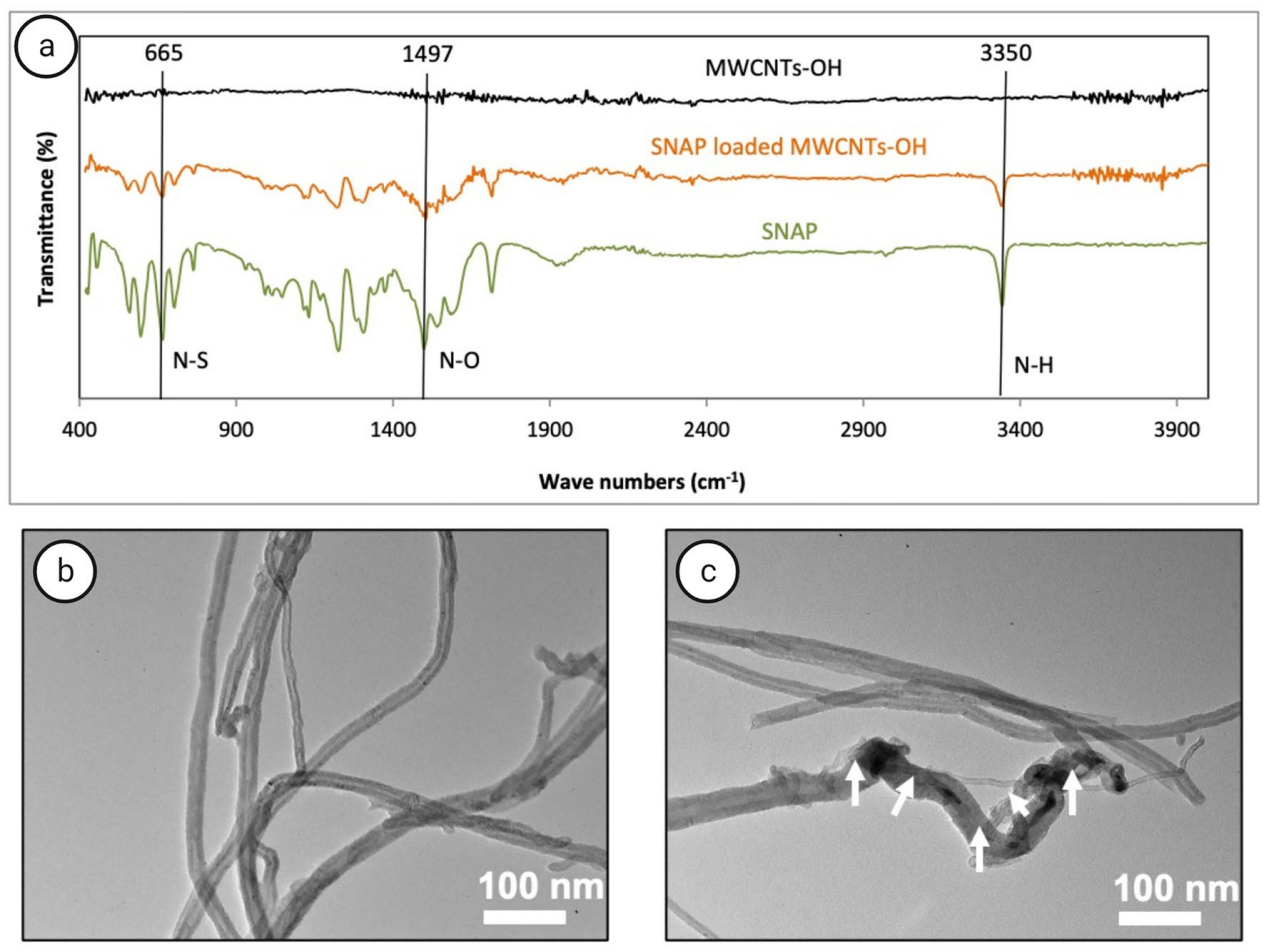
Characterization of ~ 5 w/w % SNAP loaded in MWCNTs. (**a**) FTIR spectra of SNAP, SNAP loaded MWCNTs-OH and MWCNTs-OH. TEM inspection of (**b**) MWCNTs and (**c**) SNAP loaded MWCNTs. White arrows indicate loaded SNAP. Scale bars: 100 nm.

The FTIR spectrum of pure MWCNTs-OH, SNAP loaded MWCNTs-OH and SNAP are presented in Fig. 3a. According to these results, while there is no significant peak at the MWCNTs-OH spectrum, SNAP and SNAP loaded MWCNTs-OH represent similar peaks attributed to N–O stretching at 1497 cm^−1^, N–H stretching at 3350 cm^−1^ and N–S stretching at 665 cm^−1^^18,19^. These observations confirm the loading of SNAP in MWCNTs-OH. For the pure MWCNTs-OH, there are small peaks between 3500 to 3800 cm^−1^ which could be due to the presence of an OH group on the surface of nanotubes. Similar peaks are observed in SNAP loaded MWCNTs-OH, which reveals that OH bond on nanotubes didn't change by loading of SNAP. Therefore, it could be concluded that loading of SNAP in MWCNTs-OH are due to physical bonds rather than a chemical reaction.

TEM images show the intertwined, long and cylindrical morphology of MWCNTs with a visible wall structure (Fig. 3b). In contrast to this non-loaded MWCNTs, the entrapment of some particles (SNAP crystals) inside the lumen of MWCNTs is visible in Fig. 3c, which is consistent with previous studies^20^. In addition, it can be concluded that the loaded SNAP are stored inside the lumen of the MWCNTs since MWCNTs were washed after loading with SNAP to remove any SNAP residue between MWCNTs.

### Improved NO controlled release from 3D printed SDVGs

Previous studies indicated that doping of 10% (w/w) SNAP into the polymeric matrices such as silicone-polycarbonate-urethane is the optimum percentage allowing a slow dissolution of SNAP and NO release^21^. Doping of more than 5 and less than 10% (w/w) SNAP in the polymeric matrix slows down the release profile by storage of SNAP in its crystalline form stabilized by a strong hydrogen bonding^22^.

It has also been demonstrated that loading of 15 and 20% (w/w) SNAP in a PEG-PCL matrix results in an increased initial burst release^4^. Therefore, in this study the NO release profile from 10, 20 and 30% (w/w) of SNAP doped into a PEG-PCL matrix with a PCL topcoat was measured. In this polymeric matrix, PEG facilitates the water absorption for the dissolution of SNAP while the hydrophobic PCL portion prevents the rapid degradation of PEG. Together with the PCL topcoat, this composite coating can regulate the NO release kinetics.

**Figure 4:**
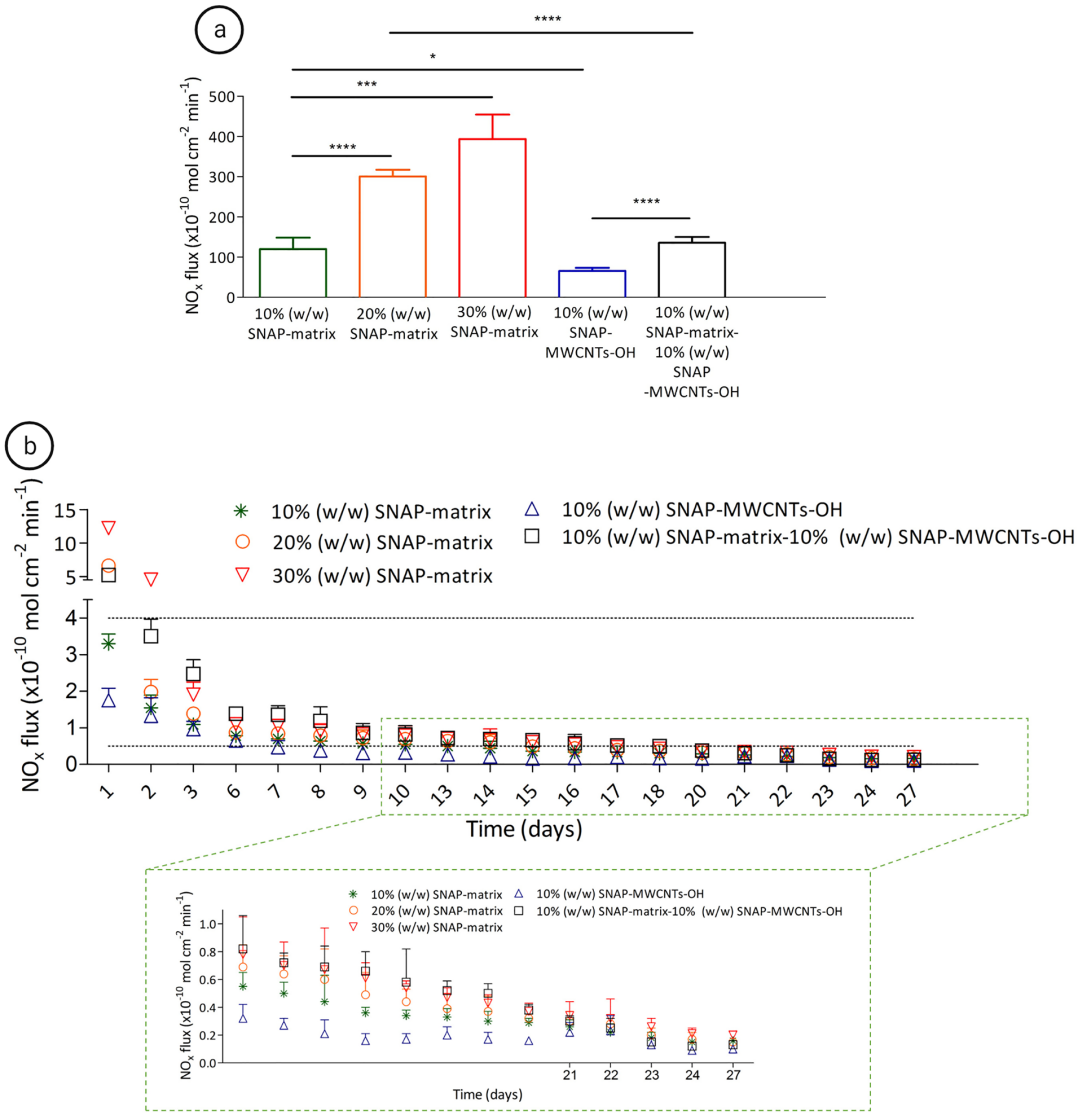
NO release profile from 3D printed grafts after coating. NO flux in PBS with 100 µM EDTA at different time points from 10%, 20% and 30% (w/w) SNAP- matrix, 10% (w/w) SNAP-MWCNT-OH and 10% (w/w) SNAP-matrix-10% (w/w) SNAP-MWCNT-OH. Part a shows the NO flux after 1 h, part b for day 1–27. The zone between black dashes represents the physiological range of NO flux (0.5–4 × 10–10 mol cm^−2^ min^−1^). Data are expressed as mean ± SD (n = 6). *P < 0.05. **P < 0.01. ***P < 0.001. ****P < 0.0001.

The NO flux from the samples with 10, 20 and 30% (w/w) SNAP-matrix, 10% (w/w) SNAP-MWCNTs-OH and 10% (w/w) SNAP-matrix—10% (w/w) SNAP-MWCNTs-OH are presented in Fig. 4.

Figure 4a,b show the NO release from the 10% (w/w) SNAP-matrix which is in the desired physiological range from 24 h until day 13. Since the prolongation of a physiological NO release, even for a few days, can be crucial for the graft function after implantation, the extension of the NO release without prolongation and/or enhancement of a burst release is an important challenge. The increase of SNAP in the polymeric matrix to 20% and 30% (w/w) prolonged the physiological NO release until day 15 and 16 in our experiments (Fig. 4b), but significantly increased the initial burst release after 1 h (Fig. 4a). Increasing the amount of SNAP in the matrix enhanced the NO release with only significantly different values in the beginning of the release period. The 10% (w/w) SNAP-matrix condition shows a significantly lower NO release compared to 20% (w/w) SNAP-matrix at 1 h and on day 1 and 3. The NO release from the 20% (w/w) SNAP-matrix condition was significantly lower on day 1, 2 and 6 when compared to the 30% (w/w) SNAP-matrix condition. These results indicate that enhancement of the SNAP concentration in the matrix prolongs the physiological NO release for a few days, but is unfortunately associated with a significant increase of the burst release.

Based on these findings and to improve the release profile, the NO release from 10% (w/w) SNAP-MWCNTs-OH doped into PEG-PCL polymeric matrix was studied. The results demonstrate a significant reduction of burst release compared with 10% (w/w) SNAP-matrix after 1 h even though the same amount of SNAP was loaded (Fig. 4a) indicating a controlling role of nanocarriers in the release kinetic. These results are consistent with previous studies showing a slowed down drug release using CNTs as a carrier^23,24^. The release of rifampicin has been prolonged by modifying the surface of a TiAl6V4 titanium alloy disc by MWCNTs, 10–200 nm in diameter, via plasma enhanced chemical vapor deposition. The antibiotic loading capacity was maximized when MWCNTs were impregnated with rifampicin showing a slow release for more than 5 days^25^. In another approach, resveratrol was loaded in CNTs and then incorporated to vascular grafts which showed a prolongation of drug release from 8 up to 30 days compared to the same grafts without CNTs^23^.

Although a significant reduction of the initial burst release could be achieved in our study by loading SNAP in MWCNTs-OH, the NO release profile remained still below the therapeutic range. Based on the results of the 20% and 30% (w/w) SNAP-matrix conditions it can be concluded that SNAP concentrations of higher than 10% (w/w) SNAP-matrix enhance and prolong the burst release. Values for 10% (w/w) SNAP-matrix remained constant in the combination experiments. Indeed, the results of 10% (w/w) SNAP-matrix in combination with 10% (w/w) SNAP-MWCNTs-OH demonstrated a controlled NO release in the physiological range for 18 days with a significant reduction of the initial burst release. The addition of 10% (w/w) SNAP-MWCNT-OH to the 10% (w/w) SNAP-matrix condition significantly enhanced the NO flux at 1 h and in the early period between day 1 to 7 and from day 13 to 20 (except day 14 and 16) compared to the 10% (w/w) SNAP-matrix condition. Loading a total amount of 20% (w/w) SNAP in matrix and MWCNTs-OH (10% (w/w) SNAP-matrix—10% (w/w) SNAP-MWCNT-OH) showed a remarkable improved NO release profile compared to 20% (w/w) SNAP in the matrix alone. A significant higher cumulative NO flux in the physiological range from day 2 until 18 was seen when 10% of the SNAP was loaded on MWCNT-OH. Comparing single days, values were significantly different at 1 h, from day 1 until 7 and on day 15 and 18 between these 2 conditions. The values for NO flux remained above the physiological range until day 18 in the 10% (w/w) SNAP-matrix—10% (w/w) SNAP-MWCNT-OH condition. It can be concluded that MWCNTs-OH led to an improvement of the NO release in the physiological range. This can be seen as a proof of concept for the role of MWCNTs-OH in improvement of release profile with the same SNAP content (Fig. 4).

Currently, there are a few studies incorporating the NO-releasing system into 3D printed medical devices. In these studies, medical devices have been coated with polymeric matrices or impregnated with the NO donor^4,26,27^. In the current study, the innovative approach refers to the incorporation of the nanocarrier-based NO releasing system into a biodegradable 3D printed graft. This state-of-the-art nanoparticle-based release system has a high potential for optimization compared to the coating and impregnation method.

### Cytocompatibility of NO releasing vascular grafts

**Figure 5:**
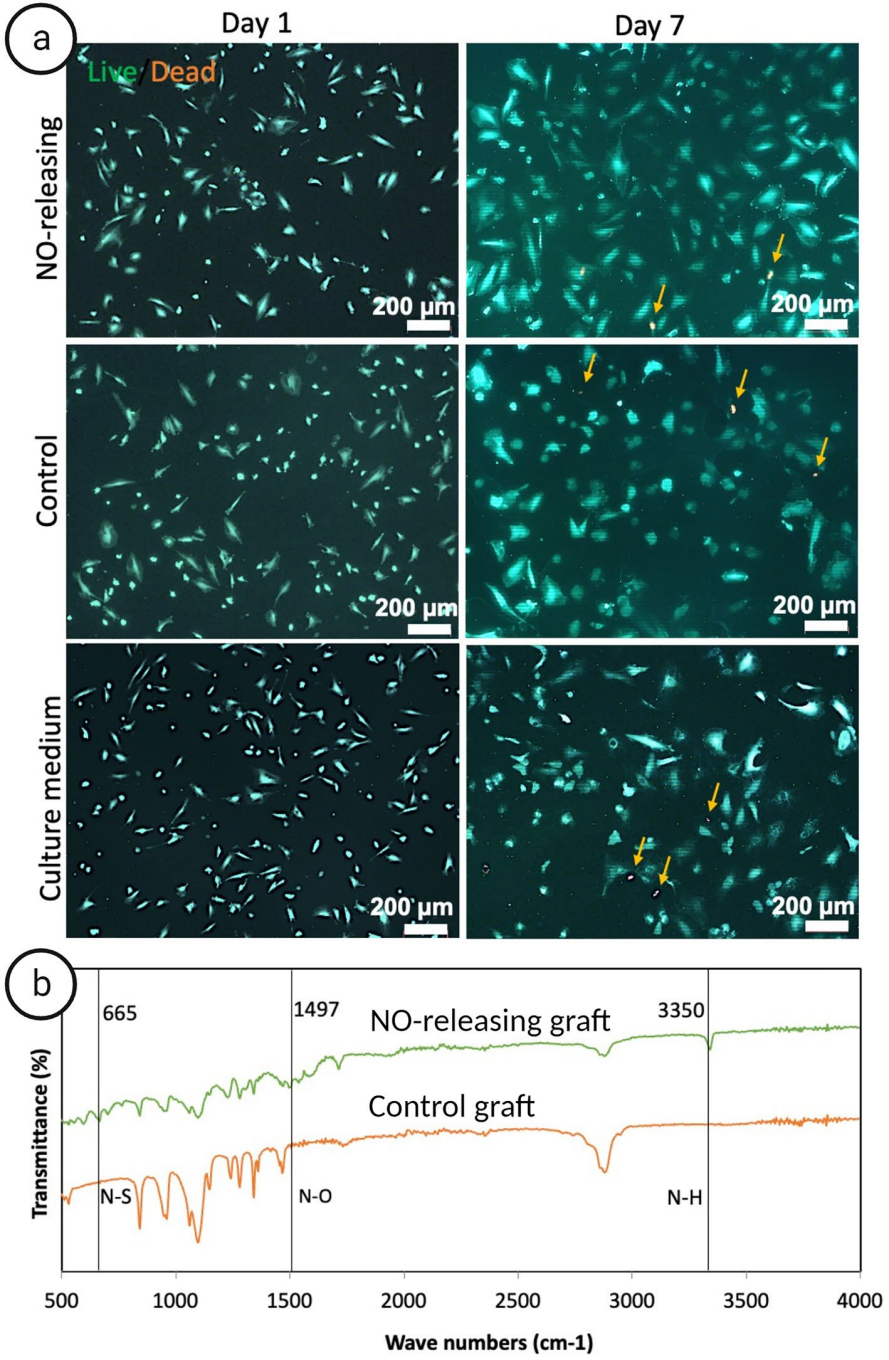
EC viability and characteristics of NO-releasing and control samples. (**a**) Live/dead fluorescence images of HUVECs incubated in direct contact with NO-releasing grafts, control grafts or culture medium. The upper row represents the cellular viability after 24 h and the lower row displays the viability after 3 days of incubation. Live cells are stained green by calcein-AM and dead cells in red by ethidium homodimer-1, respectively. Orange arrows indicate dead cells. (**b**) FTIR spectra of NO-releasing (10% (w/w) SNAP-matrix—10% (w/w) SNAP-MWCNTs-OH) and control samples (non-loaded matrix—non-loaded MWCNTs-OH) which used for biological experiments. Scale bar = 200 µm.

The cytocompatibility of NO-releasing (10% (w/w) SNAP-matrix—10% (w/w) SNAP-MWCNTs-OH) and control grafts (non-loaded matrix—non-loaded MWCNTs-OH) was evaluated by live/dead assay after direct contact with human umbilical vein endothelial cells (HUVECs). ECs presented as a layer of live cells with only single dead cells (orange arrows) in all conditions suggesting that both NO release and the presence of MWCNTs-OH in the coating layer are cytocompatible and no further residue from the solvent (toluene) present in the final grafts. As shown in Fig. 5a, HUVECs proliferated in all conditions after 3 days. This result is in line with previous studies showing no cytotoxicity for physiological NO release^12,28^ and CNTs^29,30^. For example, cardiac fibroblasts demonstrated normal growth and metabolic activity after 3 days on incubation with CNTs. FTIR analysis showed that in the NO-releasing samples, 3 representative peaks for SNAP have been visualized. They could be attributed to N–O stretching at 1497 cm^−1^, N–H stretching at 3350 cm^−1^ and N–S stretching at 665 cm^−1^ are visible while these peaks are absent in the control spectrum (Fig. 5b).

### EC proliferation, morphology and migration in response to NO releasing grafts

In situ endothelialization with EC coverage of the implanted surface is a key factor for the clinical success of SDVGs and their integration into the native tissue. NO accelerates EC proliferation and migration^31,32^. Therefore, graft surfaces with controlled NO release have the advantage of promoting initial EC migration after implantation while repopulated ECs will gradually release NO themselves. To prove this, the cellular proliferation and migration in presence of the NO-releasing grafts, control grafts and culture medium were investigated.

**Figure 6:**
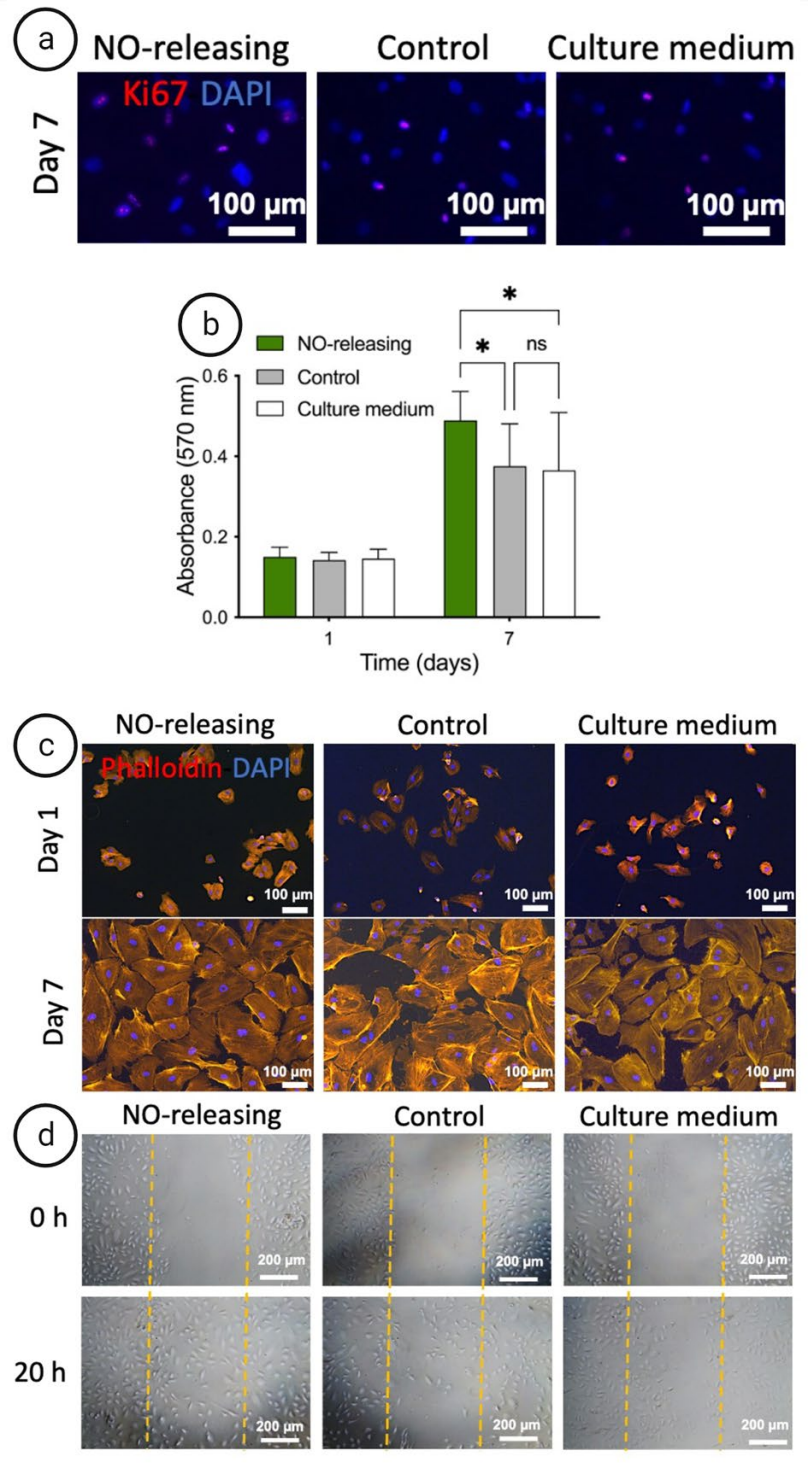
Evaluation of the NO-releasing effect on HUVEC proliferation, morphology, and migration. (**a**,**b**) Proliferation. (**a**) Immunofluorescence Ki67 staining (pink) and nucleus DAPI staining (blue) ECs incubated with NO releasing graft, control graft and culture medium after 7 days. (**b**) The obtained absorbances by MTT assay indicated a significant increase in the proliferation of HUVECs in response to NO-releasing samples while the proliferation rate was not significantly different in the presence of control samples or culture medium. (**c**) Morphology. Immunofluorescent imaging of HUVECs after 1 and 7 days of cultivation in direct contact with NO-releasing grafts, control grafts and culture medium. F-actin was stained in red (phalloidin) and nuclei were stained in blue (DAPI). (**d**) Migration. Scratch assay of HUVECs in presence of NO-releasing graft, control graft and culture medium. The monolayer of HUVECs was scratched with a 1000 µL pipette tip indicating orange dots at the beginning of the experiment (upper row) and after 20 h (lower row) of incubation. Data are shown as mean ± SD (n = 6). *P < 0.05.

The results of the Ki 67 staining (Fig. 6a) indicate a significant enhancement (P < 0.001) of EC proliferation in the NO releasing condition (48.91 ± 1.54) compared to control grafts (30.96 ± 0.75) and culture medium (32.66 ± 0.94). The MTT assay shows that the NO release from the grafts significantly enhanced the proliferation of cells compared to the control grafts, which showed similar values as incubation with culture medium (Fig. 6b). The results of cell proliferation are in accordance with the Live/Dead assay results and confirm the cytocompatibility of the samples.

To study the effect of NO-releasing SDVGs on cell morphology, HUVECs were incubated with the NO-releasing and control grafts for 7 days. The cells showed a spreading out morphology representing the proper condition for cell growth in the NO-releasing and the control conditions. Moreover, the same cell size and nuclei size in all conditions confirm a normal growth of HUVECs (Fig. 6c).

Migration of ECs from the adjacent vessel towards the site of implantation is crucial in healing of vascular grafts in situ. Figure 6d represents the scratch cell-free area at the beginning of the experiment and the migration of cells towards the wounded site after 20 h. The images display a significant enhancement (P < 0.001) of HUVEC migration after incubation with NO-releasing samples (79.43 ± 6.17). However, in presence of control grafts there is no significant difference in the motility of HUVECs (49.06 ± 5.25) compared to the migration rate in culture medium (51.47 ± 4.2) confirming the effect of NO release on EC migration. A plausible explanation for this finding is the influence of exogenous NO in the physiological range on the actin filament elongation. It has been reported that in response to NO release the vasodilator-stimulated phosphoprotein (VASP) protein concentration in the filopodia and VASP phosphorylation is increased^33^.

From the results on cell proliferation and migration it can be concluded that NO releasing samples can potentially accelerate the EC migration from the neighbor vessel towards the wounded site of the graft. This potential benefit in endothelial coverage of the implant in vivo could effectively result in an increased healing and tissue integration, which consequently improves the clinical success.

### Antibacterial properties of NO-releasing grafts

**Figure 7:**
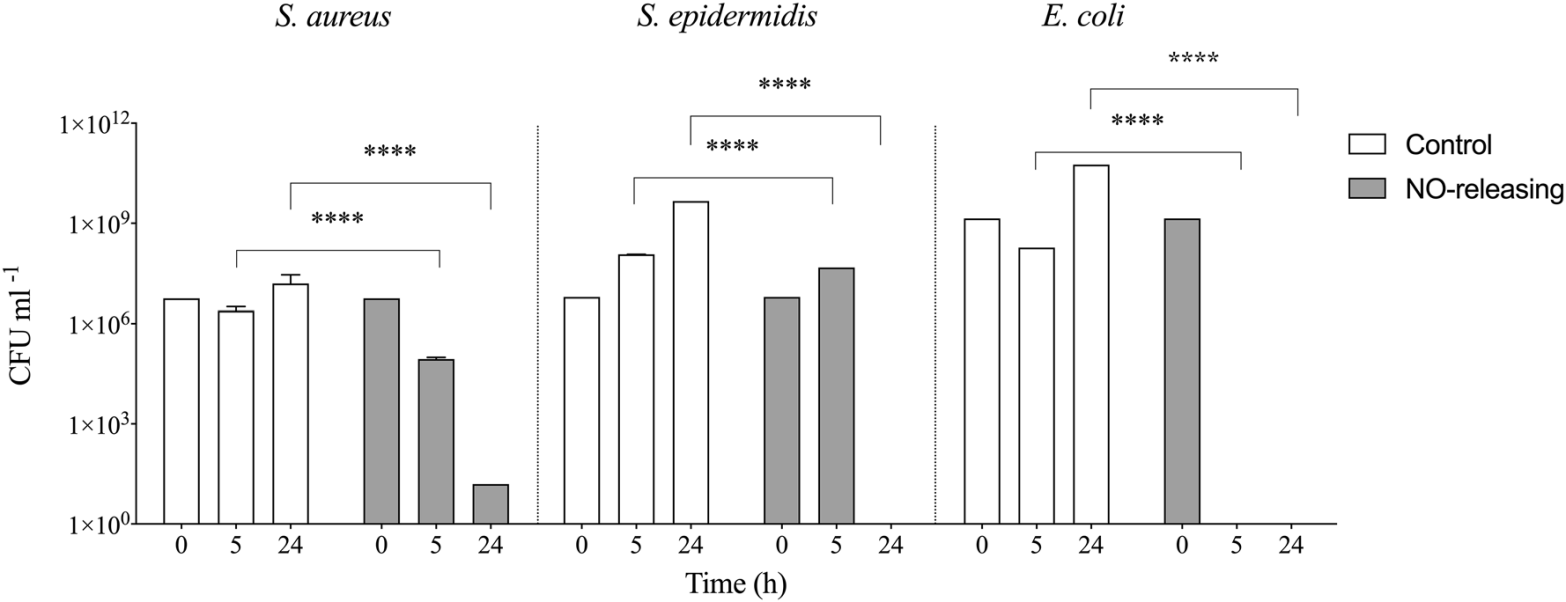
Bacterial viability. Bacterial growth after incubation with NO-releasing and control grafts of Gram positive strains (*S. aureus* and *S. epidermidis*) and the Gram negative strain (*E. coli*). The data were collected at different time points of incubation in PBS with 10% culture media. Data are shown as mean ± SD (n = 6). ****P < 0.0001.

One of the physiological roles of NO is the antimicrobial properties by deamination of DNA, lipid oxygenation in the bacterial matrix and denaturation of bacterial enzymes^34,35^. SDVGs manifest a high incidence of failure due to a high risk of bacterial infection which can be potentially reduced by NO. As a proof of concept, antibacterial properties of the NO-releasing and control grafts were studied using the most common Gram-positive and Gram-negative pathogens responsible for medical device associated infections. The results demonstrate that in presence of NO-releasing samples, the viability of both strains was significantly reduced after 5 h and 24 h of incubation. For *S. epidermidis* and *E. coli*, all bacteria were eradicated after 24 h and 5 h, respectively, while the control strains proliferated until the steady-state level (Fig. 7). However, for *S. aureus* the number of bacteria was reduced significantly from 10^7^ CFU mL^−1^ to 10 CFU mL^−1^. This slightly higher resistance of *S. aureus* to NO release is explained by its higher pathogenicity in agreement with prior results^4^.

In conclusion, this study demonstrated that SNAP was successfully loaded on MWCNTs-OH confirmed by stretching bonds in the FTIR analysis. The incorporation of loaded MWCNTs-OH into a 3D printed SDVGs enabled a significantly improved physiological NO release from the nano-composite coating with reduced initial burst release. According to the in vitro results, this coating potentially accelerates re-endothelialization and tissue regeneration in vivo while reducing the risk of implant failure by inhibiting the risk of infection. This controlled NO release system presents new opportunities for a broad range of implantable medical devices and tissue-engineered scaffolds and forms the basis for the development of future controlled NO release systems.

## Materials and methods

### Materials

*N*-Acetyl-d-penicillamine (NAP), sodium nitrate, vanadium (III) chloride (VCl_3_), sulfanilamide, *N*-(1-naphthyl) ethylene diamine dihydrochloride (NEDD), PCL (Mn = 80,000), BioUltra grade PEG (Mn = 4000), dimethyl sulfoxide (DMSO), and thiazolyl blue tetrazolium were purchased from Sigma-Aldrich (St. Louis, MO). Poly caprolactone (PCL) filament (Mn ≈ 100,000 g/mol, white, diameter 1.75 mm) under the commercial name Resomer^®^ C was supplied by Evonik Corp. (USA). Hydroxyl (OH) functionalized multiwalled carbon nanotubes (MWCNTs, OD: 8–15 nm, L: 10–50 µm) were purchased from IoLiTec (Germany). Sulfuric acid (H_2_SO_4_), hydrochloric acid (HCl), ortho-phosphoric acid (H_3_PO_4_), methanol (MeOH), tetrahydrofuran (THF), toluene, tryptic soy broth (TSB), Luria broth (LB), and sodium nitrite (NaNO_2_) were obtained from Merck (Darmstadt, Germany). Endothelial cell basal medium, FCS and supplement pack endothelial cell obtained from PromoCell (Heidelberg, Germany). Calcium and magnesium free Dulbecco’s phosphate buffered saline (PBS, pH 7.4), Dulbecco’s modified Eagle’s medium (DMED), fetal bovine serum (FBS) and penicillin/streptomycin were purchased from Gibco (Scotland, UK). Live/Dead viability/cytotoxicity kit, Alexa Fluor™ 594 Phalloidin, and DAPI were supplied by Thermo Fisher, Invitrogen™ and BD Pharmingen, respectively.

### 3D-printing of SDVGs

The stereolithography (STL) file of the tubular SDVG were designed in Autodesk fusion 360 software to be 10 mm in height and 4.5 mm in internal diameter with 0.1 mm wall thickness. Next, the STL file was converted to a G-code by slicing the CAD model in the Cura 4.6.1 software (Ultimaker, Netherlands) and imported to the Fused Filament Fabrication (FFF) 3D printer, Zaribo 420 MK3s (Caribou3d Research & Development, Germany). The PCL filament was extruded through a 80 °C nozzle with 100 μm internal diameter.

### Synthesis of SNAP

SNAP was synthesized according to a previously established protocol^12,28^. Briefly, 1 g of NAP was dissolved in 25 mL of methanol. Then, 15 mL of 1 M HCl, 500 μL of H_2_SO_4_ (98%), and 724.5 mg of NaNO_2_ were added to the NAP solution. The solution was stirred in the dark for 15 min and kept on ice without stirring for 45 min for precipitation of SNAP green crystals. Next, the SNAP crystals were filtered and rinsed with ice-cooled DI water and dried by lyophilization. The final dried crystals were kept in the darkness at − 20 °C.

### Effect of solvent polarity and MWCNT concentration

To study the effect of solvent types and MWCNTs concentration on the SNAP loading capacity, 10 mg of SNAP was incubated with THF, MeOH and toluene and 25, 50 and 100 mg of MWCNTs. The SNAP loaded MWCNTs were washed three times with iced cooled DI water to remove the excess of non-loaded SNAP and then lyophilized. Finally, the SNAP loading capacity of MWCNTs was compared with MWCNTs-OH.

### Characterization of SNAP loaded MWCNTs

To investigate the loading of SNAP in the MWCNTs FTIR was performed. Before the analysis, SNAP loaded MWCNTs-OH were rinsed with ice cooled milli-Q water three times and lyophilized, to remove any non-loaded SNAP. The FTIR spectra of SNAP, SNAP loaded WMCNTs and pure MWCNTs were measured from 400 to 4000 cm^−1^ with a Bruker Vertex 70 spectrometer (Bruker Optics, Evere, Belgium). Since MWCNTs-OH have no nitrogen and SNAP molecules have two nitrogen atoms in their structure, CHNS elemental analysis by an EA1108 Elemental Analyzer (Carlo Erba Instruments, Italy) was performed in this study to verify the SNAP loading in MWCNTs-OH. To study SNAP loaded MWCNTs and MWCNTs by transmission electron microscopy (TEM), the nanotubes were attached to (300 mesh TEM) support grids (Ted Pella, PA, US) by first adhering a tiny piece (~ 1 mm) of a carbon SEM-support sticker to the grid, and subsequently touching the dry nanotubes with the carbon sticker. By this approach, when observing nanotubes in the TEM, the imaged parts of the nanotubes were actually ‘free floating’ in the column of the TEM. In this way nanotubes were imaged with a transmission electron microscope (JEOL Ltd., Tokyo, Japan), operated at an accelerating voltage of 80 kV and equipped with a 11MPxl Quemesa camera (EMSIS GmbH, Münster, Germany). Images were taken at pixel sizes of 0.61 and 0.27 nm.

### Coating of 3D printed SDVGs

Different coating solutions were prepared by 10, 20 or 30 (w/w)% SNAP-loaded matrix, 10 (w/w) % SNAP-loaded MWCNTs-OH and 10% (w/w) SNAP-matrix—10% (w/w) SNAP-MWCNT-OH. According to the results of our previous study, the matrix was made from a 1:1 mass ratio of a PEG-PCL solution which was covered with a layer of PCL topcoat^12^. The control grafts were coated with the same coating solution with non-loaded MWCNTs-OH together with a PCL topcoat layer. For coating, 200 mL of coating solution was injected inside the rotating grafts in two steps. After solvent evaporation from the coating layer, tubes were dip coated in a PCL solution of 100 mg dissolved PCL in 1 mL toluene to form a topcoat layer.

### FTIR and SEM characterization of SDVGs

FTIR spectra of selected NO releasing (10% (w/w) SNAP-matrix—10% (w/w) SNAP-MWCNTs-OH) and control (non-loaded matrix—non-loaded MWCNTs-OH) grafts for biological studies was measured from 400 to 4000 cm^−1^ with a Bruker Vertex 70 spectrometer (Bruker Optics, Evere, Belgium). Coated and non-coated 3D printed tubes were mounted on SEM-supporting stubs with silver paint, and sputter-coated with 10 nm chromium with a Leica ACE600 coating machine (Leica-Microsystems GmbH, Vienna, Austria). The specimens’ morphology was visualized using a SE-detector of a Zeiss Sigma scanning electron microscope (Carl Zeiss Microscopy GmbH, Jena, Germany) at the acceleration voltage of 2 kV.

### NO release measurement

The grafts were immersed in 1 mL PBS with 100 µM EDTA as releasing medium and incubated at 37 °C in darkness (n = 6). At each time-point of 1 h and every 24 h, the solution was collected for NO quantification and replaced with 1 mL of fresh media. The modified Griess assay was used to measure the NO release from the samples^12,36^. Briefly, 50 µL sample and 50 µL of 1:1 mixture of 2 g sulfanilamide in 100 mL DI water with 3.44 mL H_3_PO_4_ (85%) and 0.2 g NEDD in 100 mL DI water were mixed, incubated at RT for 15 min. Next, by further incubation of the samples with 50 µL of the VCl_3_ solution, 400 mg VCl_3_ in 50 mL 1 M HCl, the nitrate content was reduced to nitrite. The absorbance was measured at 550 nm and the NO concentration was calculated using a nitrite calibration curve.

### Cell culture and evaluations

#### Human umbilical vein endothelial cell (HUVEC) culture

HUVECs (C-12208, PromoCell, Germany) originating from a cell line in passage (P) 4–6 were cultivated in EC growth medium MV2 with 1% penicillin–streptomycin solution in humidified air containing 5% CO_2_ at 37 °C. The culture medium was replaced with fresh medium every two days and cells were monitored for proliferation and absence of contamination every day.

#### Cytotoxicity

To visualize the viability of HUVECs after incubation with the NO-releasing and control samples, a LIVE/DEAD Viability/Cytotoxicity Kit was used (Invitrogen, USA). This staining visualizes simultaneously vital cells (green) with calcein-AM and dead cells (red) by ethidium homodimer-1, indicating interacellular esterase activity and loss of plasma membrane integrity, respectively. 1 × 10^4^ cells were seeded on gelatin coated glass coverslips (D = 10 mm) and cultivated in 24-well plates in the presence of the 1/20 of NO-releasing graft, control graft and culture medium. At both time points, cells were stained with 1 µL calcein-AM and 2 µL ethidium homodimer-1 in 1 mL PBS after washing. After 15 min incubation at 37 °C, the cells were observed with an Axiovert 200 M microscope (Zeiss, Oberkochen, Germany). All images (15–20 per sample) were acquired using the Zeiss proprietary software Axiovision (Rel. 4.8.2).

#### Cell proliferation

To study the proliferation of cells in contact with NO-releasing and control grafts, the Ki67 staining and MTT assay were performed.

1 × 10^4^ cells at P5 were seeded on gelatin coated glass coverslips (D = 10 mm) and incubated in culture medium with either 1/20 of NO releasing or 1/20 of control grafts or without sample. Cells were incubated in 24 well plates at 37 °C and the culture medium was changed every two days. After 7 days, cells were rinsed with PBS, fixed with 4% paraformaldehyde (10 min, RT), washed with PBS and permeabilized with 0.3% triton X-100 (20 min, RT). After blocking with 3% bovine serum albumin (BSA) solution (1 h, RT), Ki67 antibody (Anti-Mo/Rt Ki-67, Invitrogen, USA) (1/100 diluted in BSA) was incubated with cells for 1 h at RT. Subsequently, cells were washed with PBS and incubated with the secondary antibody (Alexa flour 568 goat anti-rat IgG, Invitrogen, USA) (1/400 diluted in BSA) for 1 h at RT. After PBS washing, cells were incubated with DAPI (5 min, RT), washed with PBS and mounted. Images were obtained at RT with an fluorescence Axiovert 200 M microscope (Zeiss, Oberkochen, Germany) with LD Plan-Neofluar objective lenses at 20 × (NA 0.4) magnification equipped with a camera (AxioCam MRc 5; Carl Zeiss). The acquisition was performed with the AxioVision software version 4.8 (Carl Zeiss).

For the MTT assay, HUVECs at P5 were seeded in 12 well plates with the density of 6 × 10^4^ cells per well. Cells were cultivated in 1 mL medium, medium in presence of the 1/20 of NO releasing sample or medium and the control specimen. Cell population was assessed at 24 h and 7 days by 3-(4,5-dimethylthiazol-2-yl)-2,5-diphenyltetrazolium bromide in PBS. At each time point, the culture medium was replaced with 300 µL of 5 mg/mL MTT solution and incubated for 4 h at 37 °C in darkness. After aspirating the MTT solution, 150 µL of DMSO were added to each well and shaken for 30 min. The absorbance of the solution was measured at 570 nm and plotted.

#### Cell morphology

1 × 10^5^ HUVECs were seeded on gelatin coated glass coverslips and incubated with 1/20 of the NO-releasing and control grafts. At days 1 and 7, cells were rinsed with PBS, fixed with 4% paraformaldehyde, washed with PBS and permeabilized with 0.5% Triton X-100 in PBS. Then, specimens were rinsed with 3% BSA solution and incubated in Alexa Fluor™ 594 Phalloidin solution (25 µL/1 mL PBS) for 20 min and DAPI solution (25 µL/1 mL PBS) for 5 min to stain cytoskeleton and nucleus, respectively. The stained cytoskeleton in red and nucleus in blue were visualized with an Axiovert 200 M microscope (Zeiss, Oberkochen, Germany) equipped with a camera (AxioCam MRc 5; Carl Zeiss). Image acquisition was performed with the AxioVision software version 4.8 (Carl Zeiss).

#### Cell migration

As encouragement of host EC migration into the implant surface can enhance the clinical success, the wound healing assay was performed to study the effect of NO-release on the EC migration rate. HUVECs were seeded in 24-well plates (n = 12) at a density of 1 × 10^4^ cells/well and incubated at 37 °C. After confluence, a scratch was made with a 1000 μL pipette tip, the media was aspirated, cells were rinsed twice with PBS and fed with fresh medium in the presence of 1/20 of the NO-releasing graft, control graft and culture medium. The scratch, as a model for the graft’s edges, was monitored with an Axiovert 25 inverted microscope (Carl Zeiss NV-SA, Belgium) at the beginning and after 20 h of scarification.

### In-vitro antimicrobial investigation

To study the antibacterial properties of the specimens, single colonies of Gram-positive (*S. aureus*, ATCC 8325-4 and *S. epidermidis*, ATCC 149900) and Gram-negative (*E. coli*, ATCC 25922) bacteria were inoculated in TSB and LB, respectively. After an overnight culture at 37 °C, the bacteria were washed 3 times with PBS and the concentration was adjusted to 1 × 10^7^ CFU mL^−1^ by OD measurement (600 nm). Each sample was incubated in 2 mL of PBS + 10% growth media under shaking at 37 °C. At each time points of 0, 5 h and 24 h, 100 µL from each NO-releasing sample or control sample containing tube of bacteria was collected, serially diluted and plated for counting of colony forming units (CFUs).

### Statistical analysis

All data were expressed as mean ± standard deviation (SD). One-way or two-way analysis of variance (ANOVA) was performed followed by Tukey post-tests. The t-test was used in addition when comparing conditions. The NO release over time in the physiological range was evaluated by comparing the values of each condition on each day and by calculating the area under the curve. P < 0.05 was considered significant.

## Supplementary Information

Supplementary Table 1.

## Data Availability

The datasets generated during and/or analyzed during the current study are available from the corresponding author upon reasonable request.
