# Supplementary material for: Carbon nanotubes as a nitric oxide nano-reservoir improved the controlled release profile in 3D printed biodegradable vascular grafts
Source: Sci Rep. 2023 Mar 22;13:4662. doi: 10.1038/s41598-023-31619-3 (PMC10033655; doi:10.1038/s41598-023-31619-3)
Supplement: Supplementary file 1 — Supplementary Table 1. [file 41598_2023_31619_MOESM1_ESM.docx]

## Supplementary table

| **Table S1. Results of the CHNS elemental analysis of SNAP loading on MWCNTs.** | | | |
| --- | --- | --- | --- |
| **MWCNTs (mg)** | **SNAP (mg)** | **Solvent** | **%N** |
| ***Functionalization*** | | | |
| 25 (NF) | 10 | Toluene | 2.07 ± 0.84 |
| 25 (F) | 10 | Toluene | 5.00 ± 0.67 |
| ***Solvent polarity*** | | | |
| 25 (F) | 10 | Non-polar (Toluene) | 5.00± 0.68 |
| 25 (F) | 10 | Polar (THF) | 0.37 ± 0.01 |
| 50 (F) | 10 | Polar (THF) | 0.50 ± 0.10 |
| 50 (F) | 10 | Polar (MeOH) | 0.30 ± 0.05 |
| ***Amount of MWCNT*** | | | |
| 25 (F) | 10 | Toluene | 5.00 ± 0.67 |
| 50 (F) | 10 | Toluene | 2.40 ± 0.45 |
| 100 (F) | 10 | Toluene | 1.50 ± 0.90 |
| F: functionalized, NF: non-functionalized | | | |
